# Ammonium transporter 1 increases rice resistance to sheath blight by promoting nitrogen assimilation and ethylene signalling

**DOI:** 10.1111/pbi.13789

**Published:** 2022-02-24

**Authors:** Xian Xin Wu, De Peng Yuan, Huan Chen, Vikranth Kumar, Seong Min Kang, Baolei Jia, Yuan Hu Xuan

**Affiliations:** ^1^ College of Plant Protection Shenyang Agricultural University Shenyang China; ^2^ Division of Plant Sciences University of Missouri Columbia MO USA; ^3^ Liaoning Province Shiyan High School Shenyang China; ^4^ School of Bioengineering State Key Laboratory of Biobased Material and Green Papermaking Qilu University of Technology (Shandong Academy of Sciences) Jinan China; ^5^ 26729 Department of Life Sciences Chung‐Ang University Seoul South Korea

**Keywords:** AMT1, sheath blight, resistance, nitrogen use efficiency, rice

## Abstract

Sheath blight (ShB) significantly threatens rice yield production. However, the underlying mechanism of ShB defence in rice remains largely unknown. Here, we identified a highly ShB‐susceptible mutant *Ds‐m* which contained a mutation at the ammonium transporter 1;1 (AMT1;1) D^358^N. AMT1;1 D^358^N interacts with AMT1;1, AMT1;2 and AMT1;3 to inhibit the ammonium transport activity. The *AMT1 RNAi* was more susceptible and similar to the *AMT1;1 D^358^N* mutant; however, plants with higher NH_4_^+^ uptake activity were less susceptible to ShB. *Glutamine synthetase 1;1* (*GS1;1*) mutant *gs1;1* and overexpressors (*GS1;1 OXs*) were more and less susceptible to ShB respectively. Furthermore, *AMT1;1* overexpressor (*AMT1;1 OX*)*/gs1;1* and *gs1;1* exhibited a similar response to ShB, suggesting that ammonium assimilation rather than accumulation controls the ShB defence. Genetic and physiological assays further demonstrated that plants with higher amino acid or chlorophyll content promoted rice resistance to ShB. Interestingly, the expression of ethylene‐related genes was higher in *AMT1;1 OX* and lower in *RNAi* mutants than in wild‐type. Also, ethylene signalling positively regulated rice resistance to ShB and NH_4_^+^ uptake, suggesting that ethylene signalling acts downstream of AMT and also NH_4_^+^ uptake is under feedback control. Taken together, our data demonstrated that the AMT1 promotes rice resistance to ShB via the regulation of diverse metabolic and signalling pathways.

## Introduction

Rice sheath blight disease (ShB), caused by *Rhizoctonia solani* Kühn (*R. solani*), significantly threatens worldwide rice cultivation (Molla *et al*., 2020). It is estimated that the yield reduction caused by ShB ranged from 8 to 50%, based on disease severity, crop stage of disease infection and environmental conditions (Savary *et al*., 2000). All known examples of ShB resistance are due to a quantitative trait that is controlled by multiple genes in rice, namely QTLs (quantitative trait loci). Many QTLs have been identified based on resistance to *R. solani* in different rice cultivars, some of which have been mapped and functionally characterized (Li *et al*., 1995; Richa *et al*., 2016, 2017). The underlying molecular mechanisms of rice resistance to ShB have been extensively investigated. *PR* (pathogenesis‐related) genes are known to be significant contributors to plant defence. Specifically, the PR5 family gene *OsOSM1* was confirmed to improve rice resistance against ShB (Xue *et al*., 2016). Similarly, overexpression of the ethylene (ET) biosynthetic gene *OsACS2* results in enhanced ShB resistance (Helliwell *et al*., 2013). A recent genome‐wide association study (GWAS) demonstrated that the F‐box protein ZmFBL41 interacts and degrades ZmCAD (a lignin biosynthesis enzyme) to inhibit ShB resistance (Li *et al*., 2019). Previously, we identified that IDD14 and IDD13 activate *PIN1a* to promote rice resistance to ShB (Sun *et al*., 2019a, 2020) and DEP1 interacts with IDD14 to negatively regulate rice defence to ShB ( Liu *et al*., 2021a). Our previous study demonstrated that brassinosteroids (BRs) are negative regulators of ShB resistance in rice, whereas ET can enhance the resistance. RAVL1, a key transcription factor of BR signalling, directly activates BR and ET signalling‐related genes to modulate the rice immunity to ShB (Yuan *et al*., 2018). Previous studies demonstrated that transcription factors such as *OsWRKY4*, *13*, *30* and *80* enhance ShB resistance in rice (John Lilly and Subramanian, 2019; Peng *et al*., 2012, 2016; Wang *et al*., 2015). Later, we proposed that OsWRKY53 functions as a negative regulator in rice resistance to ShB (Yuan *et al*., 2020). In a more recent study, we identified that rice sugar transporters *SWEET11* and *SWEET14* negatively and positively regulate the rice resistance to ShB, respectively (Gao *et al*., 2018; Kim *et al*., 2021). Also, DOF11 promotes rice resistance to ShB by direct activation of *SWEET14* (Kim *et al*., 2021).

Previous studies have revealed that high doses of nitrogen (N) fertilizer can cause a significant increase in the occurrence of ShB (Molla *et al*., 2020). However, limited N supply will restrict growth and yield in plants. Therefore, it is of great significance to identify genes with high nitrogen use efficiency (NUE), high resistance and high yield under low N conditions. Paddy‐soil grown rice uses ammonium (NH_4_^+^) as the primary nitrogen source (Britto *et al*., 2001). There are at least ten *OsAMTs* that mediate NH_4_^+^ uptake in the rice genome. Three polarly localized members OsAMT1;1, OsAMT1;2 and OsAMT1;3 of the AMT1 subfamily are the primary ammonium transporters, specifically under low NH_4_^+^ conditions. The three members are cooperatively responsible for NH_4_^+^ uptake in rice (Konishi and Ma, 2021). Overexpression of *OsAMT1;1,* a key transporter of NH_4_^+^ increases NUE, develops larger plants, increases yield under limited NH_4_^+^ and is involved in the rice defence response against pathogens (Pastor *et al*., 2014; Ranathunge *et al*., 2014), suggesting that NH_4_^+^ uptake plays important roles in the balance of rice growth and defence. However, the detailed underlying molecular mechanisms remain unknown.

Here, the role of AMT1‐mediated NH_4_^+^ uptake and subsequent assimilation in rice defence to ShB was investigated. The data suggest that N‐metabolites rather than NH_4_^+^ regulate rice defence. In addition, the genetic and physiological experiments demonstrated that chlorophyll and amino acids, but not γ‐ Amino acid butyric acid (GABA) metabolism, positively regulate ShB resistance. N‐dependent gene expression analysis in *AMT1;1 OX* and *AMT1 RNAi* identified that ethylene biosynthetic and signalling genes were under the control of AMT1. Interestingly, ethylene signalling controls the NH_4_^+^‐dependent *AMT1* induction via feedback regulation. Taken together, our analyses provide insight into the molecular mechanism of N transport and assimilation in ShB resistance in rice and identify the new signalling pathways by which rice modulates ShB defence under NH_4_^+^ fertilizer.

## Results

### *AMT1;1* *D^358^N* mutant accumulates less NH_4_^+^ and is more susceptible to ShB

**Figure 1:**
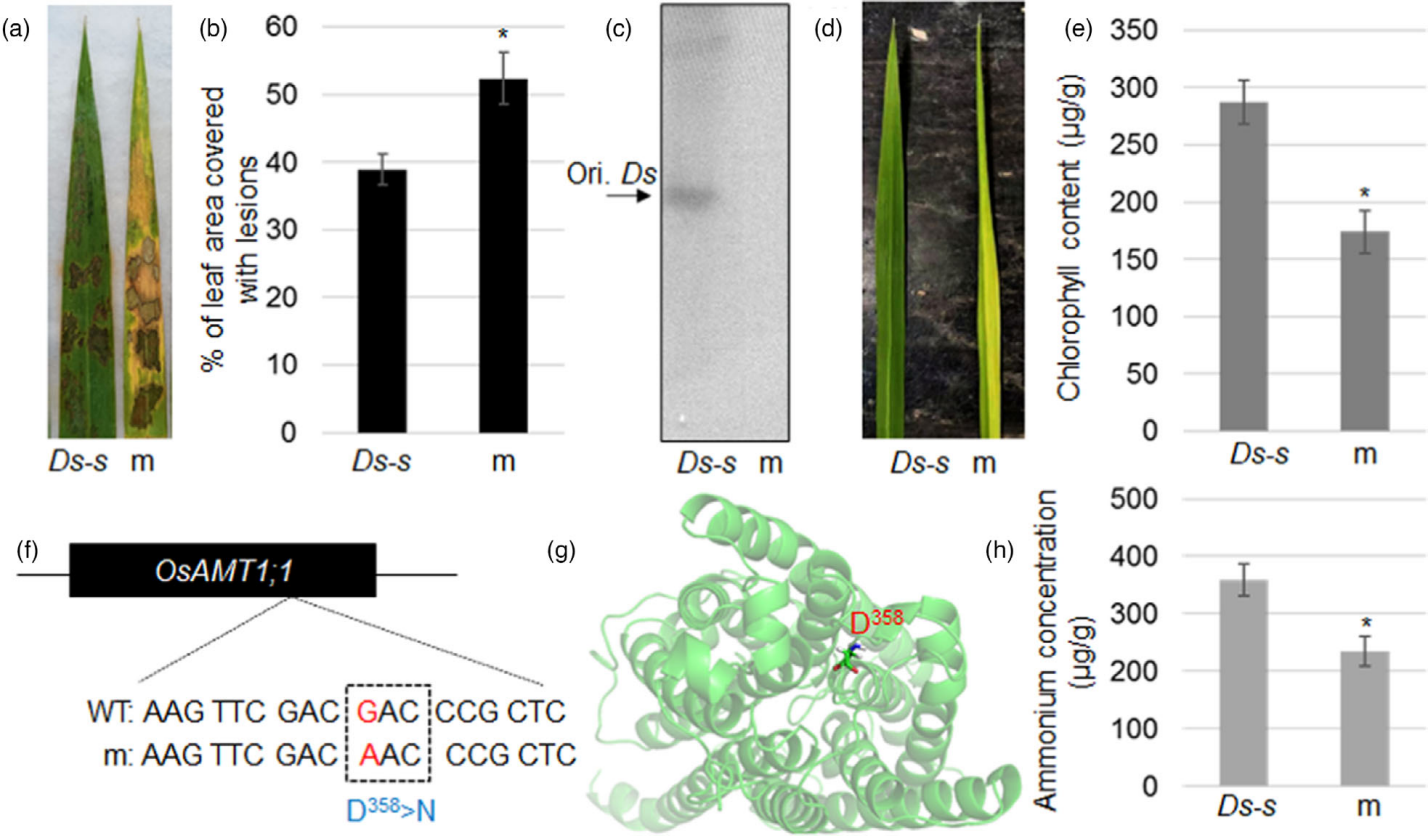
AMT1;1 D^358^N inhibits chlorophyll accumulation, NH_4_^+^ uptake and rice resistance to ShB. (a) Leaves of the starter line for regeneration (*Ds‐s*) and mutant (m) were inoculated with *R. solani* AG1‐IA and photographed after infection for 48 h. (b) The lesion area on the leaves shown in (a) was analysed. Data represent the means standard error (SE) (n > 10). (c) *Ds* insertion was examined by Southern blot analysis. *GUS* DNA fragment was used as a probe. Ori. *Ds* indicates the original *Ds* insertion site in *Ds‐s*. (d) Leaf morphology of *Ds‐s* and m plants. (e) Chlorophyll content from *Ds‐s* and m plants was determined. (f) The mutation site of in *OsAMT1;1* coding region was identified. The D^358^ was replaced with N in m plants. (g) AMT1;1 structure display, and position of D^358^ at the TM helix. (h) The NH_4_^+^ content was calculated in *Ds‐s* and m plant roots. Significant differences at the *P* < 0.05 level are indicated by stars.

Previously, we have isolated ShB‐resistant and susceptible genes via *Ds* transposon tagging in rice mutants (Sun *et al*., 2019b). Among the lines, one more susceptible mutant (*Ds‐m* (m)) was identified in this study (Figure 1a,b). However, Southern blot analysis indicated that *Ds‐m* did not contain the *Ds* fragment (Figure 1c). Since *Ds‐m* leaves showed a pale green phenotype and accumulated less chlorophyll compared to wild‐type (WT) (Figure 1d,e), *AMTs*, *GS/GOGAT*, and chlorophyll biosynthetic and catabolic genes were sequenced (data not shown). Interestingly, the sequencing results identified that G^1072^of *AMT1;1*was changed to A, which results in amino acid replacement from aspartic acid (D)^358^ to asparagine (N) (Figure 1f). D^358^ is located at the transmembrane helix (Figure 1g), and *AMT1;1 D^358^N* mutants accumulated less NH_4_^+^ than did in WT plant roots (Figure 1h).

**Figure 2:**
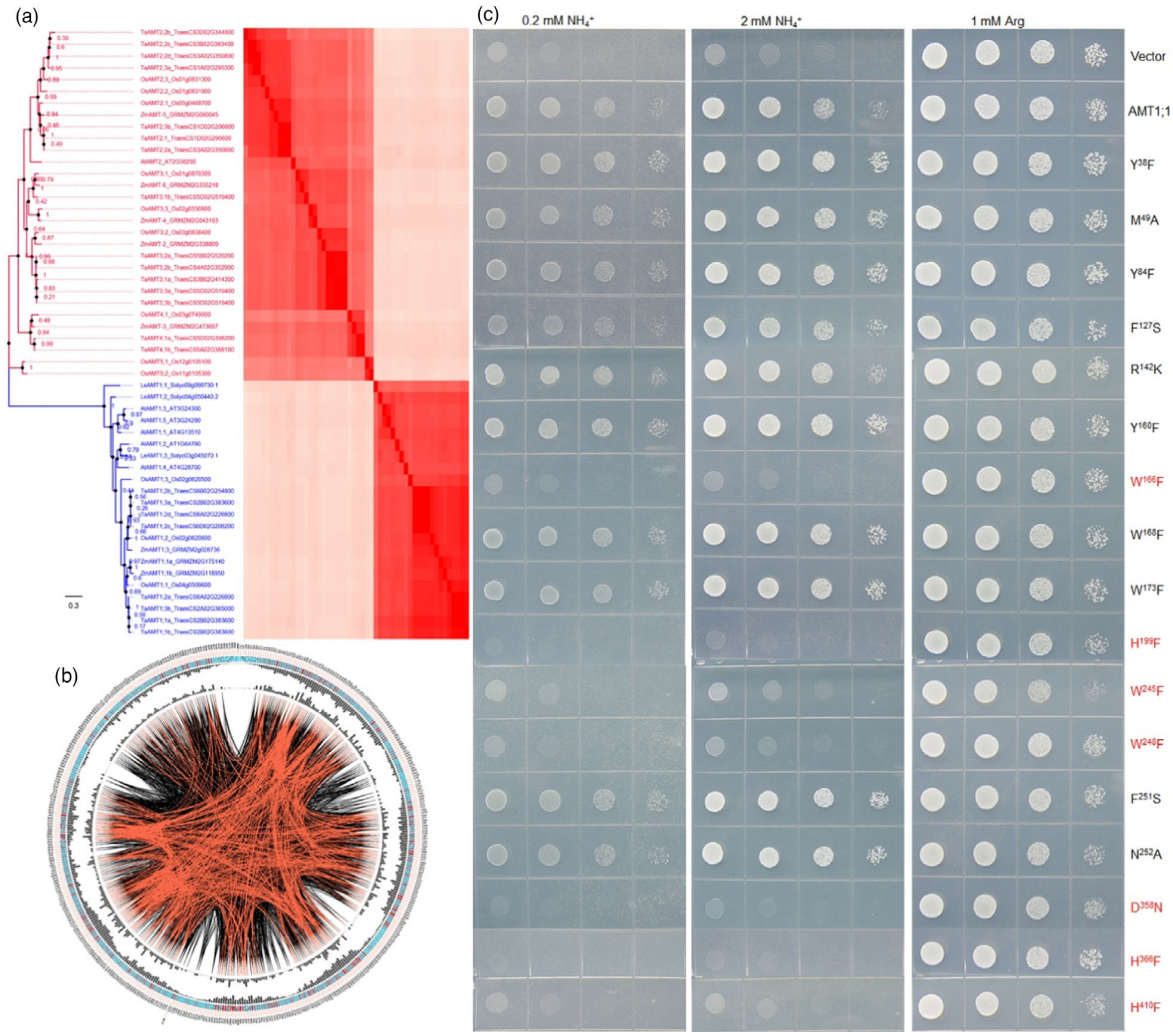
Function of the conserved residue of AMTs. (a) Phylogenetic tree for AMTs was generated using MEGA 7.0. The percentage of replicate trees in which the associated taxa clustered together in the bootstrap test (1000 replicates) is shown next to the branches. Heat map representation of protein sequence identities of the AMTs analysed by ClustalW. (b) Network analysis of conserved and coevolving residues from plant AMTs. The circular network shows the connectivity of coevolving residues. The coloured square boxes in the circle indicate MSA position conservation (highly conserved positions are shown in red and less conserved positions in blue). The second and third circles show the proximity mutual information (MI) and cumulative MI (cMI) values as histograms facing inward and outward respectively. In the centre of the circle, the edges that connect pairs of positions represent significant MI values (>6.5), with red lines indicating the highest MI scores (top 5%), black lines indicating midrange scores (between 70 and 95%) and grey lines indicating the lowest scores (the remaining 70%) as defined by MISTIC. (c) Except for AMT1;1 mutants Y^38^F, M^49^A, Y^84^F, F^127^S, R^142^K, Y^160^F, W^168^F, W^173^F, F^251^S and N^252^A, other mutations (W^166^F, H^199^F, W^245^F, W^248^F, D^358^N, H^366^F and H^410^F) in AMT1;1 led to loss of NH_4_^+^ transport activity. Empty vector (pDRf1) and AMT1;1 were used as the negative and positive controls respectively.

Before investigating the function AMT1;1 D^358^N, we examined the conservation of plant AMT members and conserved residues via heat map analysis of amino acid similarity. The results showed that the AMTs were highly conserved (Figure 2a). Next, the protein sequence of OsAMT1;1 was used as the reference sequence to determine consensus sites, the conservation of the primary sequences of AMTs by creation of multiple sequence alignments (MSAs) and the conserved residues are presented in Table S1 and Figure 2b. Interestingly, D^358^ was included within the recognized conserved amino acids. The *Δmep123* yeast strain that is defective in NH_4_^+^ transport was used to test the amino acid transport activity. The yeast growth assay indicated that AMT1;1 W^166^F, H^199^F, W^245^F, W^248^F, D^358^N, H^366^F and H^410^F failed to transport NH_4_^+^, while the other conserved residue mutations did not affect AMT1;1 activity (Figure 2c). These results demonstrated that D^358^N mutation affected AMT1;1 activity.

### AMT1;1 D^358^N inhibits AMT1;1, AMT1;2 and AMT1;3 NH_4_^+^ transport activity to affect rice resistance to ShB

**Figure 3:**
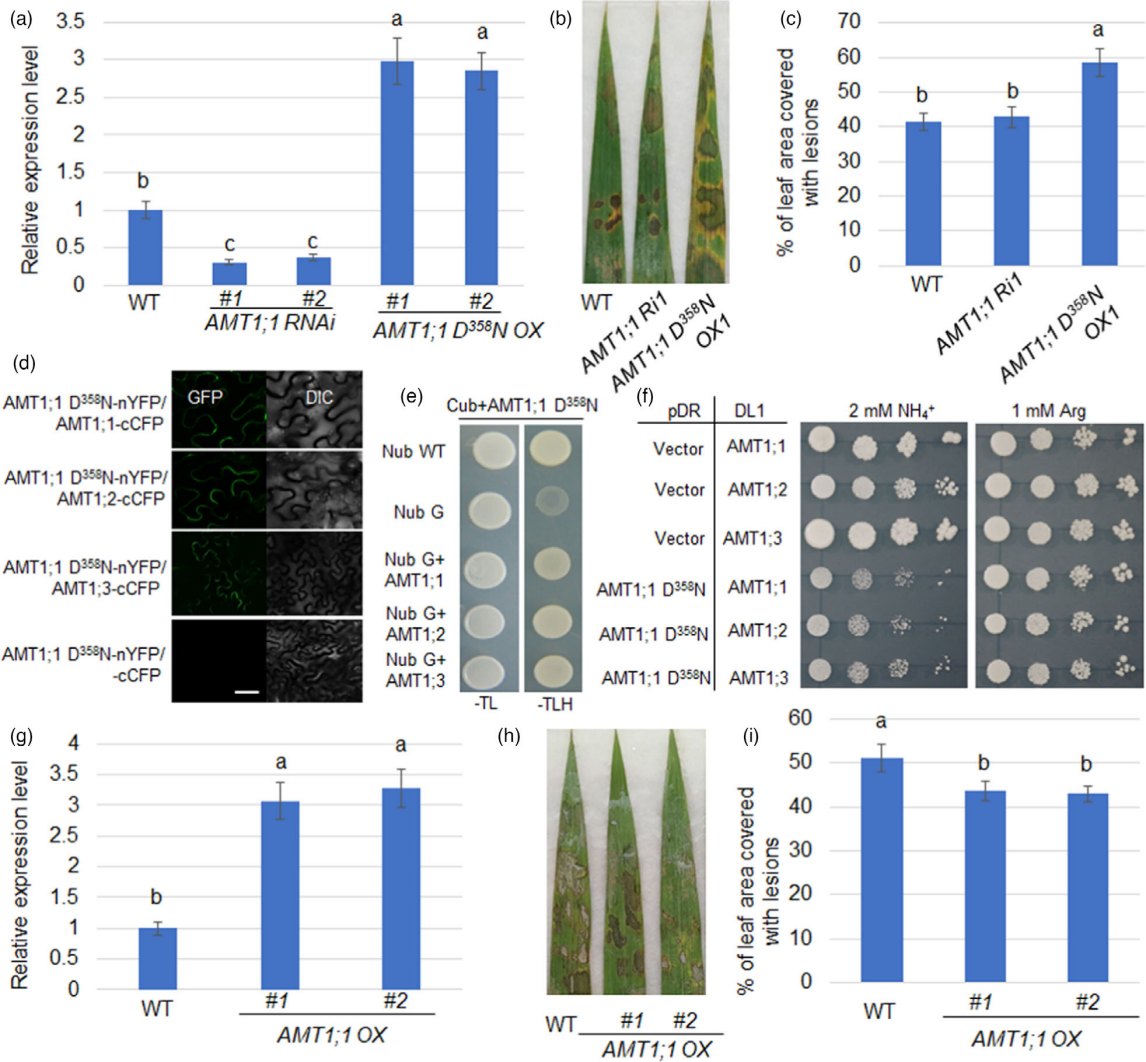
AMT1;1 D^358^N inhibits AMT1;1, AMT1;2 and AMT1;3 NH_4_^+^ transport activity to affect rice resistance to ShB. (a) qRT‐PCR was performed to analyse the expression level of *AMT1;1* in *AMT1;1 D^358^N OXs* (*OX1, OX2*)*, AMT1;1 RNAi* (*Ri1*, *Ri2*) and wild‐type. Sample mRNA levels were normalized to those of *Ubiquitin* mRNA. Error bars represent means ± SE (*n* = 3). (b)The leaves of wild‐type, *AMT1;1 RNAi* (*Ri1*) and *AMT1;1 D^358^N OX1* were infected with *R*. *solani* AG1‐IA and photographed after 3 days of infection. Six leaves from each line were analysed, and the experiments were repeated three times. (c) The lesion area was calculated from the leaves shown in (b). Data represent means standard error (SE) (*n *> 10). (d) Reconstitution of the YFP fluorescence from AMT1;1 D^358^N‐nYFP‐AMT1;1‐cCFP, AMT1;1 D^358^N‐nYFP‐AMT1;2‐cCFP or AMT1;1 D^358^N‐nYFP‐AMT1;3‐cCFP (left, fluorescence channel; right, bright field). Co‐expression of AMT1;1 D^358^N‐nYFP‐cCFP was used as the negative control. Bars = 20 μm. (e) The interaction of AMT1;1 D^358^N and AMT1;1, AMT1;2 or AMT1;3 was tested via split‐ubiquitin yeast two‐hybrid assays. (f) Yeast growth assay was performed using *Δmep123* strain to detect the NH_4_^+^ transport activity of AMT1;1, AMT1;2 or AMT1;3 with co‐expression of AMT1;1 D^358^N. *Δmep123* was also used to generate the strain DL1 by integrating *AMT1s* into the *Gap1* gene to generate *ΔGap1*::*AMT1;1*, *ΔGap1*::*AMT1;2* and *ΔGap1*::*AMT1;3*. pDR‐f1 vector was used to express *AMT1;1 D^358^N*. The yeast cells were grown on solid yeast nitrogen‐based (YNB) medium at pH 5.2 containing 2% glucose, 2 mM ammonium chloride or 1 mM arginine as sole N source, at 28°C for 3 days. (g) The *AMT1;1* expression level was identified in *AMT1;1 OXs* (*OX1*, *OX2*) by qRT‐PCR. Sample mRNA levels were normalized to those of *Ubiquitin* mRNA. Error bars represent means ± SE (*n* = 3). (h) The leaves of wild‐type and *AMT1;1OX* were infected with *R*. *solani* AG1‐IA and photographed after 3 days of infection. Six leaves from each line were analysed, and the experiments were repeated three times. (i) The lesion scales were analysed for the *R. solani AG1‐IA‐*infected leaves shown in (h) by determination of the lesion area on the leaf surface. Data represent means ± standard error (SE) (*n *> 10). Significant differences at the *P* < 0.05 level are indicated by different letters.

Since D^358^N affects AMT1;1 function and *AMT1;1 D^358^N* susceptible to ShB, *AMT1;1 D^358^N* overexpressors (*OXs*) and *AMT1;1 RNAi* plants were examined to test the defence response. Analysis by qRT‐PCR showed that *AMT1;1* expression was higher in *AMT1;1 D^358^N OXs (OX1*, *OX2)* and significantly lower in *AMT1;1 RNAi* (*Ri1*, *Ri2*) plants compared to WT (Figure 3a). The methyl‐ammonium (MeA) uptake assay demonstrated that *AMT1;1 D^358^N OXs* and *AMT1;1 RNAi* plants (Li *et al*., 2016) were insensitive to 10 mM of toxic ammonium analog MeA compared with WT (Figure S1a, b). *R. solani* inoculation results showed that *AMT1;1 D^358^N OXs* were more susceptible than WT, while *AMT1;1 RNAi* exhibited a response similar to WT (Figure 3b). The percentage of leaf area covered with lesions was 41.3% in WT, 42.8% in *AMT1;1 RNAi (Ri1)* and 58.7% in *AMT1;1 D^358^N OX1* plants (Figure 3c), suggesting that *AMT1;1 D^358^N* rather than *AMT1;1 RNAi* plants are more susceptible to ShB.

A previous study demonstrated that AtAMT1;3 T^464^D mutant inactivates AtAMT1;1 and AtAMT1;3 function (Yuan *et al*., 2013). Therefore, the functional interaction between AMT1;1 D^358^N and AMT1;1, AMT1;2 or AMT1;3 was tested. Bimolecular fluorescence complementary (BiFC) and split‐ubiquitin yeast two‐hybrid assays showed that AMT1;1 D^358^N interacted with AMT1;1, AMT1;2 or AMT1;3 (Figure 3d,e). To test whether this interaction affects AMT1;1, AMT1;2 or AMT1;3 NH_4_^+^ transport activity, yeast growth assays were performed using the *Δmep123* strain. The stable integration of *AMT1;1*, *AMT1;2* or *AMT1;3* into the yeast genome (*Δgap1::AMT1;3*) restored the growth defect of the yeast mutant DL1. However, episomal co‐expression of *AMT1;1 D^358^N* resulted in significant inhibition of yeast growth phenotypes that demonstrated the inhibition of NH_4_^+^ transport activity by AMT1;1, AMT1;2 or AMT1;3 (Figure 3f).

The above results suggest that AMT1;1 D^358^N is a dominant‐negative mutation that inhibits AMT1;1, AMT1;2 or AMT1;3 activity. The *AMT1 RNAi* (suppression of all *AMT1;1*, *AMT1;2* and *AMT1;3*) plant (Kumar *et al*., 2020) and *AMT1;1 overexpressors* (*OXs*) response to ShB was examined. The *AMT1;1* expression level was significantly higher in *AMT1;1 OXs* (*OX1*, *OX2*) (Figure 3g), and *AMT1;1 OXs* were more sensitive to MeA (Figure S1c,d). Inoculation with *R. solani* showed that *AMT1;1 OXs* were less susceptible to ShB compared to WT (Figure 3h). The percentage of leaf area covered with lesions was 40.6% in WT, 28.9% in *AMT1;1 OX1*, and 29.4% in *AMT1;1 OX2* plants (Figure 3i). To avoid side effects from overexpression, *AMT1;1* endogenous promoter was used to drive *AtAMT1;3 T464D‐A141E* Amtrac, a high‐capacity ammonium sensor, which has higher ammonium transport activity (De *et al*., 2013). Semi‐quantitative PCR detected heterologous expression of *AtAMT1;3 T464D‐A141E* in rice (Figure S2a). The *AtAMT1;3 T464D‐A141E*‐expressing plants accumulated more NH_4_^+^ than WT (Figure S2b). Inoculation with *R. solani* demonstrated that *AtAMT1;3 T464D‐A141E‐*expressing plants were less susceptible to ShB compared to WT (Figure S2c). The leaf area covered with lesions corresponded to 41.2% in WT, 30.4% in *AtAMT1;3 T464D‐A141E‐1* and 31.3% in *AtAMT1;3 T464D‐A141E‐2* plants (Figure S2d).

### N‐metabolites, rather than NH_4_^+^ itself, regulate rice resistance to ShB

**Figure 4:**
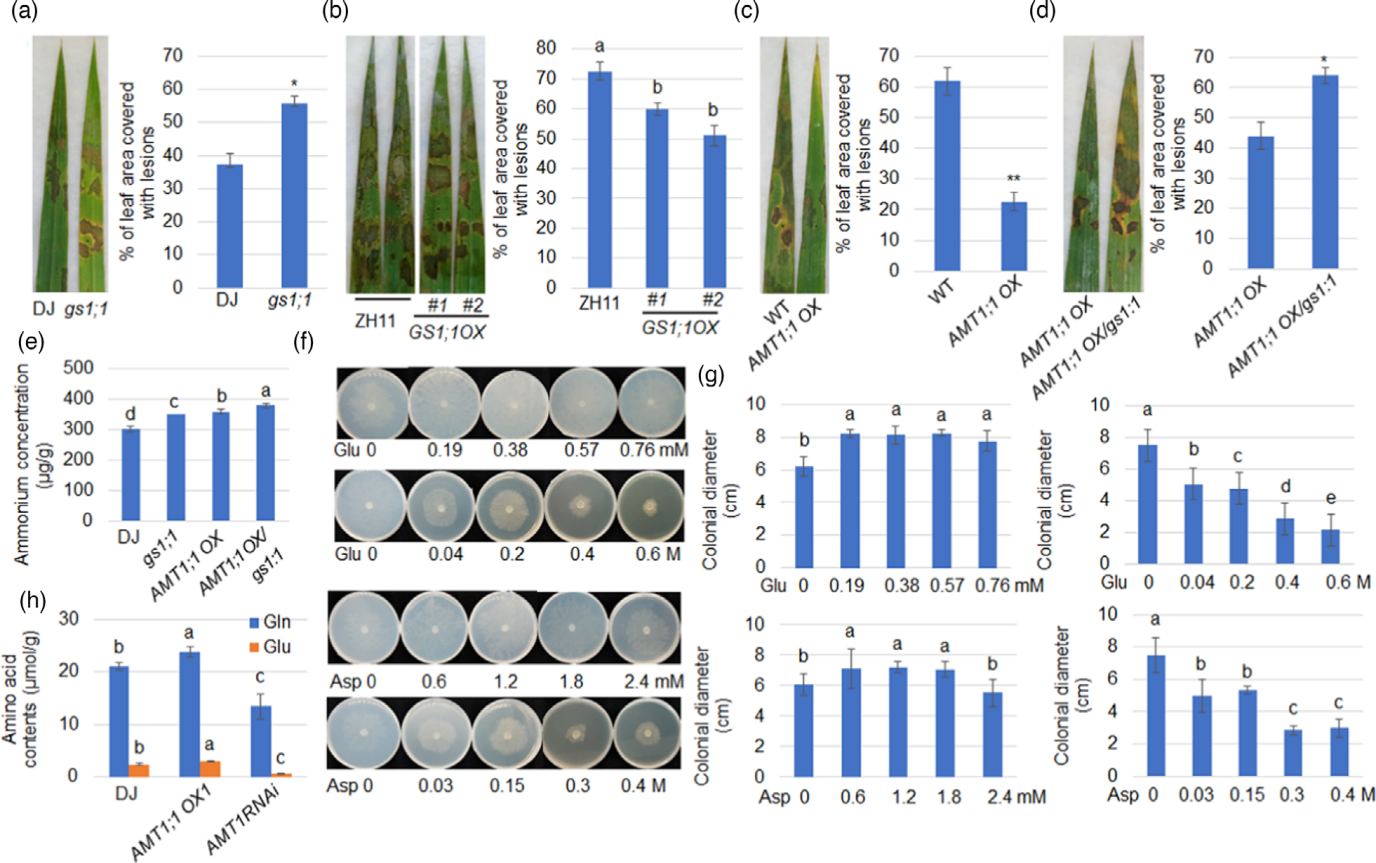
N‐metabolites rather than NH_4_^+^ regulate rice resistance to ShB. (a, b, c, d) Leaves from *GS1;1* mutant (*gs1;1*), *GS1;1* rice overexpression lines (*OX1* and *OX2*), *AMT1;1 OX1*, *AMT1;1 OX1/gs1:1* and together with their wild‐type plants were challenged with *R. solani* AG1‐IA. The corresponding statistical results of the lesion area were calculated. Six leaves from each line were analysed, and the experiments were repeated three times. Data represent the means ± standard error (SE) (*n *> 10). Significant differences at *P* < 0.05 are indicated by different letters. (e) Endogenous NH_4_^+^ levels in *gs1;1*, *AMT1;1 OX1*, *AMT1;1 OX1/gs1;*1 and wild‐type plants were measured in roots grown in 0.5 × MS for 3 days. (f, g) *R. solani* AG1‐IA was cultured on a Czapek–Dox medium with the addition of different concentrations of amino acids (glutamic acid and aspartic acid), and the colony diameter (with original cake diameter) was measured after 48 hours. The experiments were repeated at least ten times. Data represent means ± standard error (SE) (*n *> 10). Significant differences at *P* < 0.05 are indicated by different letters. (h) Glutamate and glutamine concentrations were measured from 10‐day‐old wild‐type, *AMT1;1 OX1* and *AMT RNAi* plant leaves grown in hydroponics for 4 weeks with a spectrophotometric method. Data represent the means ± standard error (SE) (*n *> 10).

Since *AMT1;1* promotes rice resistance to ShB, the role of the NH_4_^+^ and N‐metabolites in rice defence to ShB was further investigated. Glutamine synthetase 1;1 (GS1;1) is the key GS enzyme in rice (Tabuchi *et al*., 2005). The *gs1;1* mutant was more susceptible, while *GS1;1 OXs* were less susceptible to ShB compared to WT (Figure 4a,b). Furthermore, *GS1;1* expression was significantly higher in *GS1;1 OXs* than in WT (Figure S3a). To further identify whether *AMT1;1‐*mediated rice resistance to ShB requires GS1;1 activity, the genetic combination of *AMT1;1 OX* and *gs1;1* was generated. Inoculation with *R. solani* revealed that *AMT1;1 OX* was less susceptible, while *AMT1;1 OX/gs1;1* and *gs1;1* were significantly more susceptible to ShB compared to WT plants (Figure 4c,d). In addition, the NH_4_^+^ content in all four genotypes (WT, *gs1;1*, *AMT1;1 OX* and *AMT1;1 OX/gs1;1*) was analysed. The results demonstrated that *gs1;1* and *AMT1;1 OX* accumulated higher NH_4_^+^ than WT. The highest NH_4_^+^ content was detected in *AMT1;1 OX/gs1;1* plants (Figure 4e), suggesting that N‐metabolites rather than NH_4_^+^ regulate ShB resistance in rice.

To explore how N metabolism affects the interaction between rice and *R. solani*, the function of amino acids during *R. solani* growth was examined. The results indicated that some common amino acids, such as glutamate (Glu), glutamine (Gln), aspartic acid (Asp), asparagine (Asn), phenylalanine (Phe), proline (Pro) and alanine (Ala), promoted the growth of hyphae in low N concentrations, while the growth of mycelia was inhibited in high N concentrations (Figure 4f,g and Figure S3). Also, amino acid concentration measurements demonstrated that *AMT1;1 OX* mutant contained more, but *AMT1 RNAi* contains less Glu and Gln than WT plants (Figure 4h). γ‐Amino Butyric Acid (GABA) is a non‐protein amino acid that is also an important product of N metabolism. It has been reported to play key roles in a variety of physiological processes in plants (Deng *et al*., 2020). However, overexpressing glutamate decarboxylase (*GDCi‐OX*) (Figure S4a,b), an enzyme that converts glutamate to GABA, increased the susceptibility of rice to ShB (Figure S4b,c). In addition, high concentrations of GABA (10 mM) promoted hyphae growth (Figure S4d,e), suggesting that amino acids and not GABA may regulate rice resistance to ShB.

### Chlorophyll accumulation promotes rice defence in response to ShB

**Figure 5:**
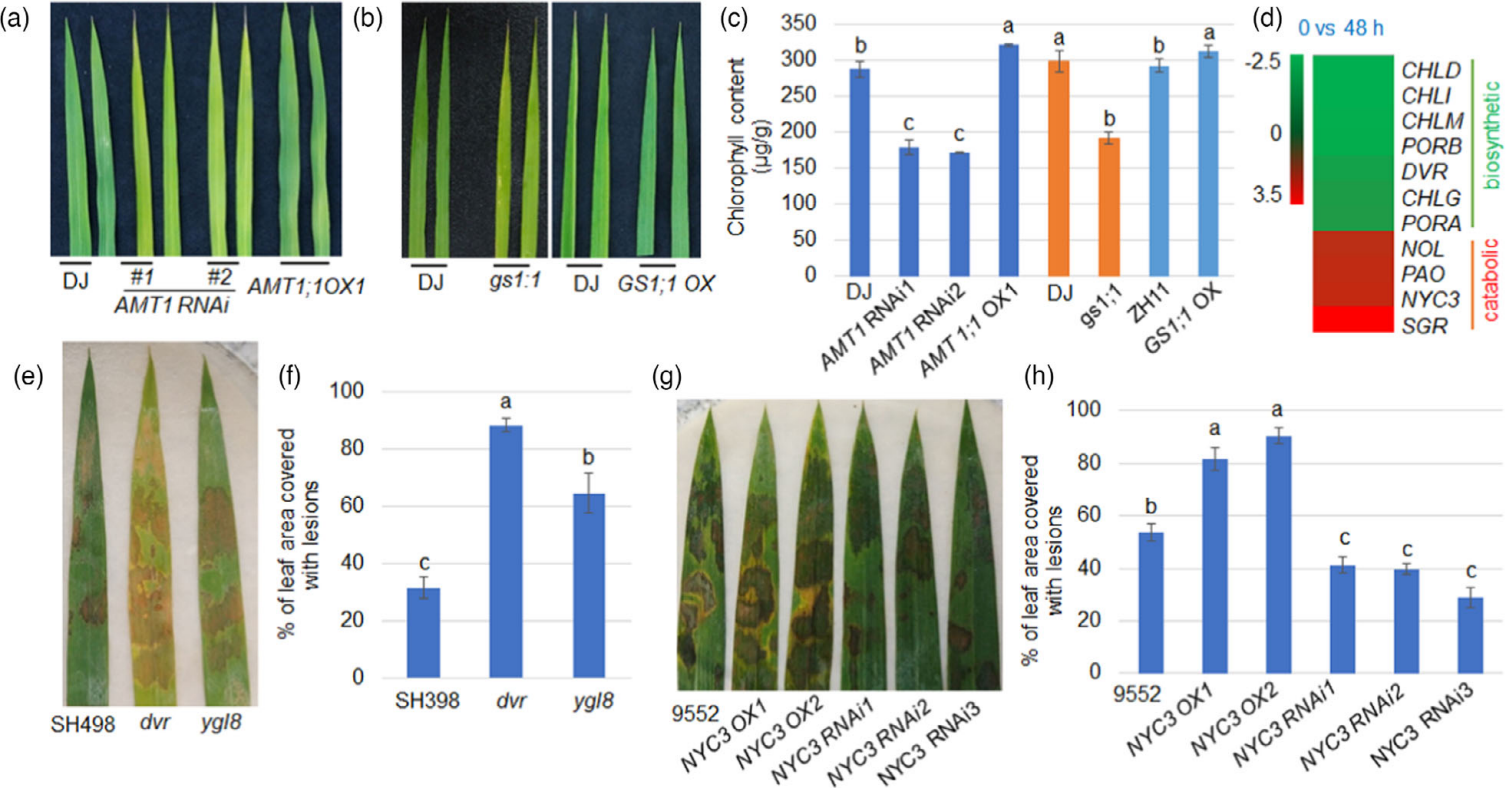
Chlorophyll accumulation promotes rice defence to ShB. (a) The leaves of *AMT1 RNAi* (*#1* and *#2*), *AMT1;1 OX* and wild‐type were photographed. (b) Leaves from *gs1;1*, *GS1;1 OX* together with their corresponding wild‐type were photographed. (c) The chlorophyll contents of *AMT1 RNAi*, *AMT1;1 OX* and *GS1;1* mutants as well as their corresponding wild‐type were determined. (d) *R*. *solani* AG1‐IA dependent (after 48 hours of inoculation) expression levels of chlorophyll biosynthesis and catabolic genes were shown in a heat map. (e) The response of chlorophyll synthesis‐related gene *DVR* and *YGL8* mutants and wild‐type Shuhui498 (SH498) to *R. solani*. (f) The lesion scales were analysed for the *R*. *solani* AG1‐IA‐infected leaves shown in (e) by determination of the lesion area on the leaf surface. (*n *> 10). (g) The response of chlorophyll catabolic gene *NYC3 RNAi* and overexpression (*OX*) plants to *R. solani*. (h) The corresponding lesion scales were analysed by determination of the lesion area on the leaf surface. Data represent means ± standard error (SE) (*n *> 10). Significant differences at the *P* < 0.05 level are indicated by different letters.

Nitrogen metabolism is involved in the synthesis of many nitrogen‐containing compounds (Baslam *et al*., 2020). A notable example is glutamate that forms 5‐aminolevulinic acid (ALA) through glutamyl tRNA reductase (GluTR) and glutamate‐1‐semialdehyde aminotransferase (GSA), functioning as the precursor to chlorophyll. Therefore, N is an important component of chlorophyll (Eckhardt *et al*., 2004). *AM* *T1 RNAi* and *gs1;1* showed a pale green leaf phenotype and contain less chlorophyll, while *AMT1;1 OX* and *GS1;1 OX* accumulated more chlorophyll (Figure 5a,b,c). Also, our previous transcriptome data showed that *R. solani* inoculation altered N uptake, assimilation and chlorophyll synthesis (*CHLD*, *CHLI*, *CHLM*, *PORB*, *DVR*, *CHLG* and *PORA*) and catabolic gene (*NOL*, *PAO*, *NYC3* and *SGR*) expression (Yuan *et al*., 2020) (Figure 5d). Inoculation of two rice mutants of chlorophyll synthesis‐related genes *DVR* (*3*,*8‐divinyl protochlorophyllide a 8‐vinyl reductase*) and *YGL8* (*yellow‐green leaf 8)* (Kong *et al*., 2016; Nagata *et al*., 2005) with *R. solani* showed that *dvr* and *ygl8* were more susceptible than WT plants (Figure 5e,f). Next, inoculation of *RNAi* and overexpression lines of chlorophyll degradation‐related gene *NYC3* (*α/β hydrolase‐fold family protein*) (Cao *et al*., 2021) demonstrated that *NYC3‐OX* was more susceptible, while *NYC3‐RNAi* was less susceptible to ShB compared to WT plants (Figure 5g,h). These results are consistent with the recently published data showing that chlorophyll content is positively correlated with ShB defence in rice (Cao *et al*., 2021).

### AMT1;1‐mediated rice resistance to ShB depends on nitrogen levels

**Figure 6:**
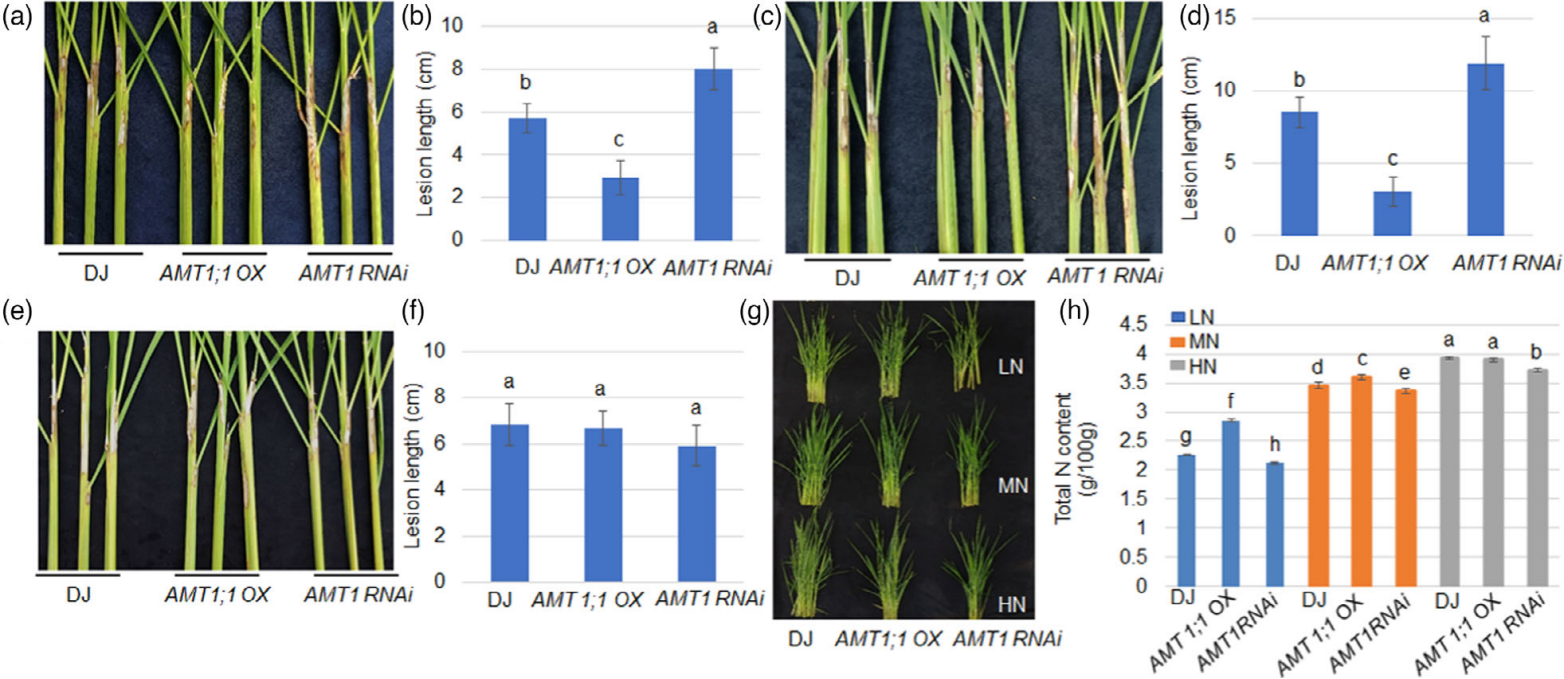
The effects of nitrogen fertilization level on AMT1;1‐mediated rice resistance to ShB. (a) Wild‐type (DJ), *AMT1;1OX* and *AMT1RNAi* plants cultured in LN supply condition (50 kg/ha) were inoculated with *R*. *solani* AG1‐IA for 14 d. (b) The lesion lengths shown in (a) were measured and statistically analysed. Data represent means ± standard error (SE) (*n *> 10). (c) Wild‐type (DJ), *AMT1;1OX* and *AMT1RNAi* plants cultured in LN supply condition (150 kg/ha) were inoculated with *R*. *solani* AG1‐IA for 14 d. (d) The lesion lengths shown in (c) were measured and statistically analysed. Data represent means ± standard error (SE) (*n *> 10). (e) Wild‐type (DJ), *AMT1;1OX* and *AMT1RNAi* plants cultured in HN supply condition (300 kg/ha) were inoculated with *R*. *solani* AG1‐IA for 14 d. (f) The lesion lengths shown in (C) were measured and statistically analysed. Data represent means ± standard error (SE) (*n *> 10). (g) Rice seedlings of wild‐type, *AMT1;1 OX* and *AMT1RNAi* plants grown under LN, MN and HN conditions for 14 days. (h) The total nitrogen from wild‐type, *AMT1;1 OX* and *AMT1RNAi* plants was determined by the Kjeldahl method. Data represent means ± standard error (SE) (*n *> 10). Significant differences at the *P* < 0.05 level are indicated by different letters.

Since AMT1;1 is an ammonium transporter, the relationship between N availability and AMT1;1‐dependent rice resistance to ShB was examined under different N fertilization conditions. *AMT1;1 OX*, *AMT1 RNAi* and WT plants were cultured under high N (urea, HN 300 kg/ha), middle N (urea, MN 150 kg/ha) and low N concentrations (urea, LN 50 kg/ha) (Liu *et al*., 2021b). *R. solani* inoculation demonstrated that *AMT1;1 OX* plants were significantly more resistant, while *AMT1 RNAi* plants were more susceptible to ShB than WT under LN (Figure 6a,b) and MN (Figure 6c,d) conditions. However, the positive effect of *AMT1;1* to rice defence against *R. solani* was eliminated under the HN fertilization conditions with no significant resistance differences among *AMT1;1 OX*, *AMT1 RNAi* and WT plants (Figure 6e,f). Total N content was also measured in *AMT1;1 OX*, *AMT1 RNAi* and WT plants that were grown under LN, MN or HN conditions. The results indicated that *AMT1;1 OX* accumulated higher, while *AMT1 RNAi* contained less N compared to WT under LN and MN conditions. Under HN conditions, *AMT1 RNAi* accumulated slightly less total N than *AMT1;1 OX* and WT plants, while *AMT1;1 OX* contained a similar level of total N compared to WT (Figure 6g,h).

### Feedback activation of NH_4_^+^ uptake by ethylene signalling is important for rice resistance to ShB

**Figure 7:**
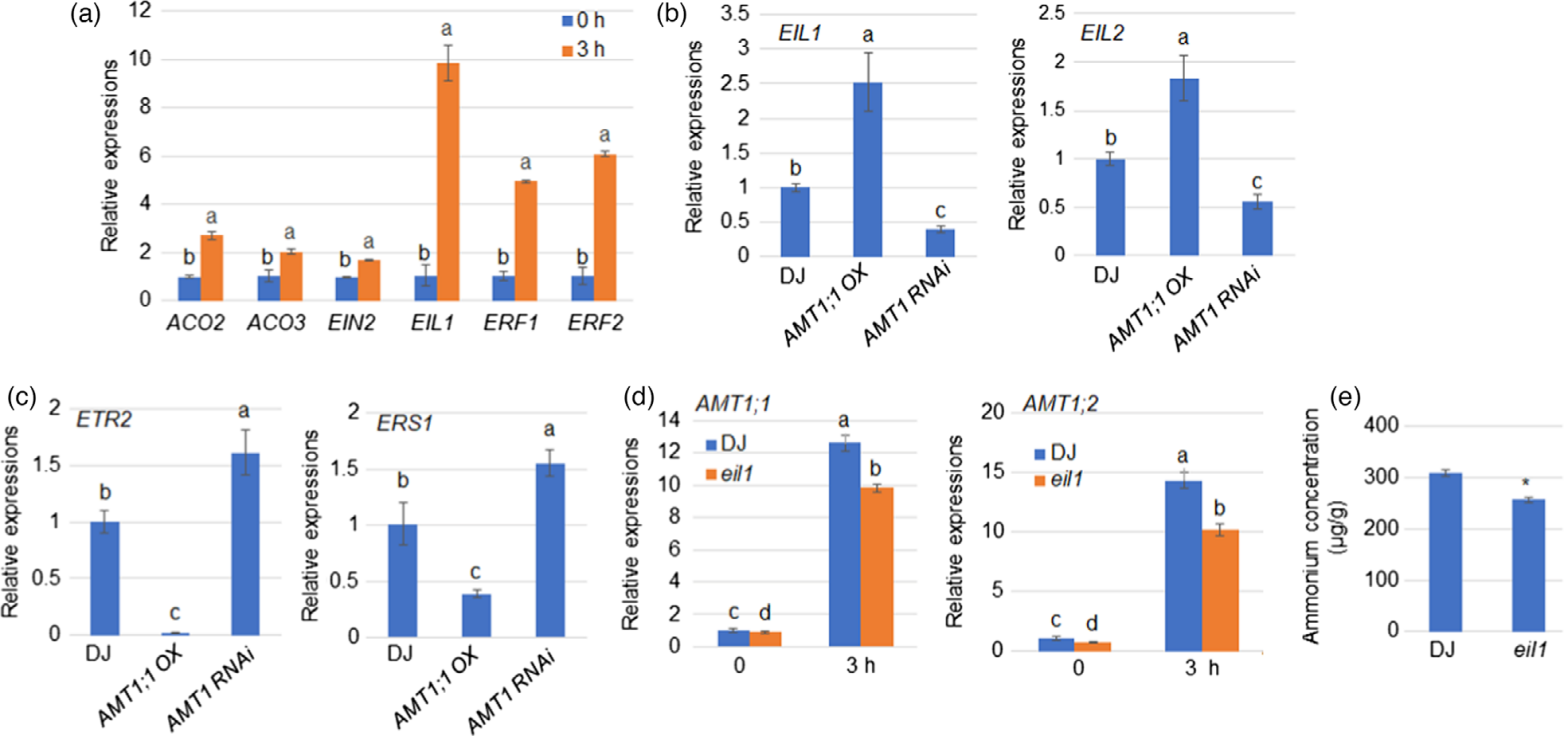
Feedback activation of NH_4_^+^ transport via ethylene signalling is important for rice resistance to ShB. (a) NH_4_^+^‐dependent expression levels *ACO2*, *ACO3*, *EIN2*, *EIL1* and *ERFs* were verified by qRT‐PCR with NH_4_^+^ treatment for 3 hours. (b) Positive ethylene signalling regulators *EIL1* and *EIL2* and (c) negative ethylene signalling regulators *ERS1* and *ETR2* expression levels were monitored in wild‐type (DJ), *AMT1;1 OX* and *AMT1 RNAi* plants grown in LN conditions for 14 days. (d) NH_4_^+^‐induced *AMT1;1* and *AMT1;2* expression levels in WT and *EIL1* mutant (*eil1*) was monitored. Sample mRNA levels were normalized to those of *Ubiquitin* mRNA. Error bars represent means ± SE (*n* = 3). (e) Endogenous NH_4_^+^ levels in WT and *eil1* were measured in roots grown in 0.5 × MS for 3 days. Error bars represent means ± SE (*n* = 20). Significant differences at the *P* < 0.05 level are indicated by different letters.

Our previous transcriptome study identified that ET biosynthesis and signalling genes were up‐regulated after NH_4_^+^ treatment (Xuan *et al*., 2013). The qRT‐PCR results verified the transcriptome data where *ACO2*, *ACO3*, *EIN2*, *EIL1* and *ERFs* were significantly induced with NH_4_^+^ treatment (Figure 7a). Previously, we identified that ET signalling positively regulates rice resistance to ShB (Yuan *et al*., 2018), suggesting that NH_4_^+^ assimilation may activate ET signalling to promote rice resistance to ShB. To test this hypothesis, *ERS1*, *ETR2*, *EIL1*, and *EIL2* expression was examined in *AMT1;1 OX*, *AMT1 RNAi* and WT plants under LN conditions. qRT‐PCR results showed that two positive ET signalling regulators *EIL1* (Mao *et al*., 2006) and *EIL2* (Yang *et al*., 2015) expression levels were higher in *AMT1;1 OX* and lower in *AMT1 RNAi* than in WT. The expression of *ETR2* (Wuriyanghan *et al*., 2009) and *ERS1* (Ma *et al*., 2014), two negative ET signalling regulators, was suppressed and induced in *AMT1;1* OX and *AMT1 RNAi*, respectively, than in WT under LN conditions (Figure 7b). However, *EIL1* and *EIL2* expression levels were lower, while *ERS1* expression was higher in *AMT1;1 OX* than in WT plants under HN. Furthermore, the *ETR2* expression level was similar in WT, *AMT1;1 OX* and *AMT1;1 RNAi* plants under HN (Figure S5). Interestingly, we found that NH_4_^+^‐mediated induction of *AMT1;1* and *AMT1;2* was inhibited in *eil1* mutants (Yuan *et al*., 2018) (Figure 7c) and that *eil1* mutants accumulated less NH_4_^+^ than WT plants (Figure 7d).

### *AMT1;1 OX* increases yield and resistance under LN conditions

**Figure 8:**
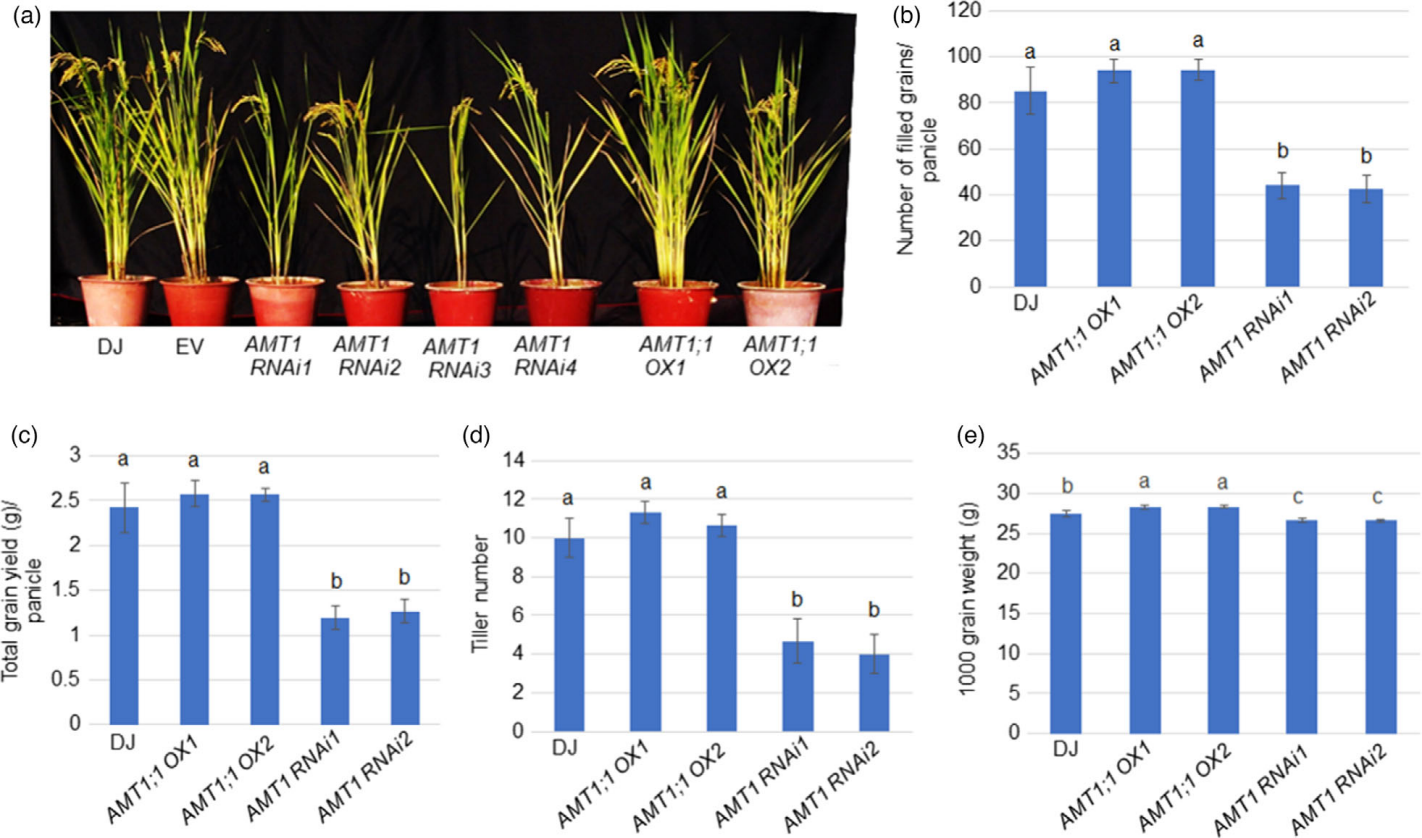
Comparison of yield index between *AMT1;1 OX*, *AMT1 RNAi* and WT plants grown in LN condition. (a) Mature plant morphology, (b) tiller number, (c) filled grains per panicle, (d) total grain yield and (e) 1000 grain weight were calculated. Data are means ± SD of 12 plants. Significant differences at the *P* < 0.05 level are indicated by different letters.

Previous studies demonstrated that overexpression of *AMT1;1* enhances NH_4_^+^ uptake and improves rice growth and yield at least under specialized N fertilization conditions (Ranathunge *et al*., 2014). Tillering is an important trait for grain yield in rice. Mature *AMT1;1 RNAi* plants developed significantly fewer tillers than in WT and *AMT1;1 OX*, while WT and *AMT1;1 OX* produced a similar number of tillers (Figure 8a,b). Furthermore, less filled grains per panicle and lower total grain yield per panicle were found in *AMT1;1 RNAi* than in WT and *AMT1;1 OX*. However, no differences were identified between WT and *AMT1;1 OX* (Figure 8c,d). The thousand‐grain weight was similar between WT and *AMT1;1 RNAi*, while *AMT1;1 OX* was higher than WT (Figure 8e).

## Discussion

ShB is one of the most important diseases, which severely affects the quality and quantity of production in rice. However, the underlying rice defence mechanisms remain largely unknown. In this study, the AMT1;1 function in rice defence to ShB was explored by analysing the roles of NH_4_^+^ and N‐metabolites as well as ET signalling during the defence process. The data illustrated that AMT1;1‐mediated NH_4_^+^ transport accelerated N metabolism and regulated subsequent NH_4_^+^‐dependent ethylene‐related gene expression to promote rice resistance to ShB under limited N fertilizer conditions, suggesting that appropriate N uptake and assimilation are necessary for rice defence activation.

### AMT1;1 D^358^N interacts with and inhibits AMT1;1, AMT1;2 and AMT1;3 to control rice defence

A pale green mutant *Ds‐m* was identified in the *Ds*‐tagging mutant pool, which was more susceptible to ShB than WT. However, Southern blot results verified that the *Ds‐m* phenotype was not caused by a *Ds* insertion. Since glutamate is the precursor of chlorophyll and forms ALA through GluTR and GSA (Eckhardt *et al*., 2004), *AMT*, *GS/GOGAT*, and chlorophyll biosynthetic and catabolic genes were sequenced in the *Ds‐m* mutant. Interestingly, *Ds‐m* contained a point mutation at G^1072^A, which resulted in the D^358^N change. Conserved residues and subsequent functional analysis revealed that D^358^ was highly conserved among plant AMTs and D^358^N replacement abolished AMT1;1 NH_4_^+^ activity. Furthermore, *AMT1;1 D^358^N OX* and *AMT1 RNAi* (suppression of *AMT1;1*, *AMT1;2* and *AMT1;3*) plants accumulated less NH_4_^+^ and were more susceptible to ShB. However, suppression of a single *AMT1;1* by RNAi did not inhibit rice resistance to ShB, suggesting that *AMT1;1 D^358^N*‐susceptible symptom may be caused by other mechanisms. A previous report demonstrated that AMT forms a homo‐ or hetero‐trimer and AtAMT1;3 T^464^D interacts and inhibits AtAMT1;1 NH_4_^+^ transport activity (Yuan *et al*., 2013). Our analysis indicated that AMT1;1 D^358^N interacted with AMT1;1, AMT1;2 and AMT1;3 and inhibited their NH_4_^+^ transport activity. AMT1;1, AMT1;2 and AMT1;3 are colocalized in the endodermis cell layer and are cooperatively responsible for the NH_4_^+^ transport in rice (Konishi and Ma, 2021), suggesting that AMT1;1 D^358^N‐mediated inhibition of AMT1;1, AMT1;2 and AMT1;3 activity can occur in *planta*. In other words, AMT1;1 D^358^N plants inhibit the function of AMT1;1, AMT1;2 and AMT1;3 to reduce rice resistance to ShB.

### N‐metabolites, but not NH_4_^+^, promote ShB resistance in rice

*AMT1;1 D^358^N* and *AMT1 RNAi* plants that contained less cellular NH_4_^+^ were more susceptible. However, *AMT1;1 OX* and *pAMT1;1‐high‐capacity Amtrac* plants that accumulated more cellular NH_4_^+^ were less susceptible to ShB, suggesting that cellular NH_4_^+^ is positively correlated with rice resistance. Next, the key glutamine synthetase gene mutant *gs1;1* was more susceptible while *GS1;1 OX* was less susceptible to ShB, despite the increased and reduced cellular NH_4_^+^ in *gs1;1* and *GS1;1 OX* respectively. These results suggest that NH_4_^+^ may not be the molecule responsible for the control of ShB resistance in rice. To definitively verify these results, *AMT1;1* was overexpressed in the *gs1;1* background. The results demonstrated that *AMT1;1 OX/gs1;1* was similar to *gs1;1* where increased cellular NH_4_^+^ was accumulated and the plants were more susceptible to ShB. Therefore, AMT1;1‐mediated rice resistance is required during the NH_4_^+^ assimilation process.

NH_4_^+^ is incorporated into the glutamine amide group by GS (Mur *et al*., 2017). A recent study reported that amino acid metabolism is an important process in the nitrogen‐mediated plant defence mechanism (Sun *et al*., 2020). Therefore, the direct role of amino acids on the growth of *R. solani* hyphae was investigated. The amino acids tested including Glu and Gln all inhibited *R. solani* growth at high concentrations. The *AMT1;1 OX* plants accumulated more Glu and Gln compared to WT. These data suggest that AMT1;1‐mediated defence may partially act via accumulation of amino acids to inhibit *R. solani* growth. However, GABA is a non‐protein amino acid that promoted *R. solani* growth even at high concentrations. Also, GABA biosynthetic gene overexpression plants *GDCi OX* were more susceptible to ShB, indicating that GABA negatively regulates rice resistance to ShB.

Glutamate is the precursor of chlorophyll (Eckhardt *et al*., 2004), and *AMT1;1 D^358^N*, *AMT1 RNAi* and *gs1;1* accumulated less chlorophyll and were more susceptible to ShB. Our transcriptome results suggested that *R. solani* infection significantly suppressed chlorophyll biosynthesis gene expression while inducing chlorophyll catabolic gene expression, suggesting a potential function of chlorophyll in rice defence. A genetic study by testing the chlorophyll biosynthetic gene *DVR* and *YGL8* mutants (Kong *et al*., 2016; Nagata *et al*., 2005), as well as chlorophyll catabolic gene *NYC3* mutant (Cao *et al*., 2021), revealed that chlorophyll content was positively correlated with rice resistance to ShB (Cao *et al*., 2021). These results suggest that AMT1‐mediated NH_4_^+^ transport and assimilation promote chlorophyll synthesis by which rice partially increased resistance to ShB.

### Ethylene signalling activates NH_4_^+^ uptake via feedback regulation to promote rice resistance to ShB

Cellular NH_4_^+^ is not only used to synthesize amino acids but also functions as a signal molecule to regulate global gene expression (Patterson *et al*., 2010). We further investigated whether other signalling pathways regulate AMT1;1‐mediated rice resistance, aside from N metabolism. Plant hormone signalling is tightly associated with rice defence to ShB (Molla *et al*., 2020). We previously identified that NH_4_^+^ treatment regulates the expression of auxin signalling genes (Xuan *et al*., 2018) and demonstrated that auxin signalling activation via exogenous IAA application improves rice resistance to ShB (Sun *et al*., 2019a), implying that NH_4_^+^ supply may modulate auxin signalling to regulate rice resistance to ShB. In addition, our previous studies identified that ET biosynthesis and signalling genes were induced by NH_4_^+^ treatment (Xuan *et al*., 2013) and that ET signalling promotes rice resistance to ShB (Yuan *et al*., 2018), suggesting that NH_4_^+^ signalling may activate ET signalling to promote rice resistance. Our analyses identified that *EIL1* and *EIL2* which activate ethylene signalling were positively regulated while *ETR2* and *ERS1*, two negative regulators of ethylene signalling, were suppressed by *AMT1;1* under the LN conditions. However, under the HN conditions, *EIL1* and *EIL2* expression levels were significantly lower while *ETR2* and *ERS1* levels were higher in *AMT1;1 OX* than in WT. These results suggest that ET signalling may be sensitive to the cellular N levels and may be associated with rice resistance to ShB. Furthermore, we identified that NH_4_^+^‐mediated induction of *AMT1* genes was inhibited in the key ET signalling gene *eil1* mutant. The *eil1* mutant accumulated less NH_4_^+^, suggesting that ethylene signalling controls NH_4_^+^ transport via feedback regulation to fine‐tune the cellular N transport and assimilation, which may be important for rice defence and growth.

### *AMT1;1 OX* increases rice resistance and NUE under limited N fertilizer

Nitrogen fertilizers supplied to rice crops are partially lost via various mechanisms including ammonia volatilization, denitrification and leaching, causing environmental concerns by polluting the atmosphere, aquatic systems and groundwater (Choudhury and Kennedy, 2005). Therefore, limiting the amounts of NH_4_^+^ applied to the fields without loss of crop yield is an important agricultural strategy in rice. Our results demonstrated that *AMT1;1 OX* plants significantly promoted rice resistance to ShB under the LN conditions. Under HN conditions, *AMT1;1 OX* accumulated similar NH_4_^+^ content compared to WT and also exhibited a similar ShB response to WT plants. As previously reported, *AMT1;1 OX* uptake more NH_4_^+^ and significantly increase yield production at least under specialized N fertilization conditions (Ranathunge *et al*., 2014). Our data also confirmed that *AMT1;1 OX* produced a relatively higher yield, suggesting that *AMT1;1 OX* increased NUE and ShB resistance in rice.

In this study, the data demonstrated that AMT1;1‐mediated increase in rice resistance was via N‐metabolite activation and ethylene signalling. This study demonstrates the precise use of nitrogen based on the underlying molecular mechanisms of N metabolism to improve yield production and immunity against ShB and other pathogens in rice.

## Materials and methods

### Plant growth and *R. solani* AG1‐IA inoculation

All of the rice plants treated with *R*. *solani* were cultured in the Shenyang Agriculture University greenhouse at 23–30°C, 80% relative humidity (RH) and 12‐h light/12‐h dark photoperiod. *Nicotiana benthamiana* plants were grown in environmental chambers at 22–24°C, 80% RH and 16‐h light/8‐h dark photoperiod for 4 weeks before use. The *R. solani* strain AG1‐IA was cultured on solid PDA (Potato Dextrose Agar) medium at 28°C in an incubator. Rice was inoculated according to previously reported methods (Cao *et al*., 2021).

### Molecular phylogenetic analysis using maximum likelihood

The amino acid sequences of AMT proteins in rice, maize, *Arabidopsis*, wheat and potato were used as bait for searching in the Uniprot database (http://www.uniprot.org/) using BLASTp. MSAs of these protein sequences were conducted using the Clustal Omega program (Larkin *et al*., 2007). Phylogenetic relationships were inferred using the maximum likelihood (ML) methods with 1,000 bootstrap iterations (Kumar *et al*., 2016).

### Ammonium uptake assay in yeast

The yeast strain *31019b* (*Δmep123*), which is defective in NH_4_^+^ absorption (Marini *et al*., 1997), was used to test the NH_4_^+^ transport activity of AMT1;1 and AMT1;1 mutants. *Δmep123* was also used to construct the strain DL1 by introducing *AMT1s* into the *Gap1* gene locus to generate *ΔGap1*::*AMT1;1*, *ΔGap1*::*AMT1;2* and *ΔGap1*::*AMT1;3*. The pDR‐f1 vector was used to express *AMT1;1 D^358^N*, following previously reported methods (Yuan *et al*., 2013). The yeast transformants were grown on solid yeast nitrogen‐based (YNB) medium at pH 5.2 containing 2% glucose (w/v), 2 mM ammonium chloride or 1 mM arginine as the sole nitrogen source.

### Vector construction and transgenic plant generation

*AMT1;1* promoter was fused to an Amtrac high‐capacity gene ORF in the *pGA1611* binary vector. The primers used for plasmid construction are listed in Table S3. *pAMT1;1:Amtrac high capacity* was transformed into Japonica rice cultivar Dongjin (DJ) calli via *Agrobacterium*‐mediated transformation method (Hiei *et al*., 1994). The *gs1;1 mutant* (PFG_3A‐09512) was obtained from a rice T‐DNA mutants collection (http://signal.salk.edu/cgi‐bin/RiceGE/) (An *et al*., 2003). The overexpression of *GS1;1* and *GDCi* was generated from the rice cultivar Zhonghua 11 (ZH11). The modified *pCAMBIA1381‐Ubi* vector was used to construct *GS1;1* and *GDCi* overexpression vectors at *Hind*III/*Kpn*I and *Hind*III/*Hpa*I respectively. The primers used for the *GS1;1* and *GDCi* overexpression vector constructions are listed in Table S4.

### Analysis of amino acid effects on *R. solani* growth

*R. solani* AG1‐IA was cultured on a Czapek–Dox medium with the addition of different concentrations of amino acids. Colonized PDA plugs (7 mm in diameter) were excised using a hole borer and transferred to the centre of the fresh media surface. These petri dishes were then cultured in a 37°C incubator for 42 hours, and the diameters of the colonies were measured. The assays were conducted repeatedly at least eight times.

### Determination of NH_4_^+^ and total N content

The NH_4_^+^ content in roots and shoots of 7‐day‐old rice seedlings was measured using an F‐kit (Roche) according to the manufacturer’s instructions (Oliveira *et al*., 2002). The total N content in rice plants was determined by the Kjeldahl method using the Hanon k1160 Automatic Kjeldahl nitrogen determinator (Shandong, China).

### RNA extraction and qRT‐PCR analysis

Total RNA was extracted from the one‐month‐old leaves from tested rice plants using TRIzol reagent (Takara, Dalian, Liaoning, China). Elimination of genomic DNA and reverse transcription reactions were performed according to the manufacturer’s instructions using the commercial kit (Takara, Dalian, Liaoning, China). qRT‐PCR analysis was performed using the CFX96 real‐time PCR system (Bio‐Rad, Hercules, CA, USA) and ChamQ Universal SYBR qPCR Master Mix (Vazyme, Nanjing, Jiangsu, China). Gene expression values were normalized against *Ubiquitin* values in the same samples. Two technical and three biological replicates were used for each analysis. The primers used for qRT‐PCR are listed in the supplemental Table S2.

### Determination of chlorophyll content

The chlorophyll content in leaves of one‐month‐old plants was determined using the ultraviolet spectrophotometer following a previously reported method (Lichtenthaler, 1987).

### Amino acid measurement

Gln and Glu content was measured using an L‐Glu analysis kit (Yamasa, Tokyo, Japan) following the manufacturer’s instructions (Hirano *et al*., 2008).

### Split‐ubiquitin yeast two‐hybrid assay

*AMT1;1*, *AMT1;2* and *AMT1;3* were fused to the N‐terminus of *Ubiquitin* through Nub vector pXN25_GW and *AMT1;1 D^358^N* was fused to the C‐terminus of *Ubiquitin* through Cub vector pMETYC_GW based on standard GATEWAY cloning protocol (Invitrogen, CA, USA). Yeast two‐hybrid assays were performed according to a previously published method (Lalonde *et al*., 2010).

### BiFC and southern blotting assays

*AMT1;1 D^358^N* was cloned into a YFP^N^ vector, while *AMT1;1*, *AMT1;2* and *AMT1;3* were cloned into CFP^C^ plasmids. The constructs were co‐transformed into tobacco leaves using *Agrobacterium* strain GV3101 (Kim *et al*., 2009). The YFP fluorescence signals were observed under a confocal microscope (Olympus FV1000, Japan) 36 to 48 hours after infiltration. Southern Blotting assay of *Ds* insertion was carried out with reference to the method described by a previous study (Xuan *et al*., 2016).

### Statistical analyses

Statistical analyses were conducted using Prism 5.0 software (GraphPad, San Diego, CA, USA) with a one‐way analysis of variance (ANOVA) for comparison of significant differences between multiple groups. Also, Student’s *t*‐test was used to compare the differences between the two groups. Differences between the groups were considered significant with at least *P* < 0.05.

## Conflict of interest

The authors declare no conflict of interest.

## Author contributions

XXW and YHX planned and designed the research. XXW, HC, DPY, VK and SMK performed most of the experiments. XXW, BLJ and DPY analysed data. XXW, BLJ and YHX wrote the manuscript. XXW, HC, DPY and VK contributed equally to this work.

## Supporting information


**Table S1**. Gateway primers used in this study.
**Table S2**. qRT‐PCR and RT‐PCR primers used in this study.
**Table S3**. Primers used in this study for the construction of plants expressing *AtAMT1;3 T464D‐A141E* driven by *AMT1;1* endogenous promoter.
**Table S4**. Primers used in this study for the construction of the overexpression vector.
**Figure S1**. Sensitivity test of *AMT1;1 RNAi* and overexpression plants to methyl‐ammonium (MeA).
**Figure S2**. *AtAMT1;3 T464D‐A141E* expression promotes rice resistance to ShB.
**Figure S3**. Verification of the effects of amino acids on *R*. *solani* growth.
**Figure S4**. Identification of the effect of GABA on rice resistance against ShB.
**Figure S5**. Expression levels of ethylene signalling genes under HN conditions in wild‐type, *AMT1;1 OX* and *AMT1;1 RNAi* plants were quantified by qRT‐PCR.
