# Supplementary material for: Ammonium transporter 1 increases rice resistance to sheath blight by promoting nitrogen assimilation and ethylene signalling
Source: Plant Biotechnol J. 2022 Feb 24;20(6):1085–97. doi: 10.1111/pbi.13789 (PMC9129087; doi:10.1111/pbi.13789)
Supplement: Supplementary file 1 — Table S1. Gateway primers used in this study. Table S2. qRT‐PCR and RT‐PCR primers used in this study. Table S3. Primers used in this study for the construction of plants expressing AtAMT1;3 T464D‐A141E driven by AMT1;1 endogenous promoter. Table S4. Primers used in this study for the construction of the overexpression vector. Figure S1. Sensitivity test of AMT1;1 RNAi and overexpression plants to methyl‐ammonium (MeA). Figure S2. AtAMT1;3 T464D‐A141E expression promotes rice resistance to ShB. Figure S3. Verification of the effects of amino acids on R. solani growth. Figure S4. Identification of the effect of GABA on rice resistance against ShB. Figure S5. Expression levels of ethylene signalling genes under HN conditions in wild‐type, AMT1;1 OX and AMT1;1 RNAi plants were quantified by qRT‐PCR. [file PBI-20-1085-s001.docx]

**Supplemental data**

**Supplemental Table 1. Gateway primers used in this study.**

| **Mutation**  **site** | **Primer name** | **Primer sequence (5'-3')**  Underlined bases are mutant bases Underlined bases are mutant bases |  |
| --- | --- | --- | --- |
| W168F | W168F-F | CACTGGATCTTCTCCGCCGATG |  |
|  | W168F-R | CATCGGCGGAGAAGATCCAGTG |  |
| W248F | W248F-F | TGGTTCGGCTTCTACGGATTCA |  |
|  | W248F-R | GAATCCGTAGAAGCCGAACCAC |  |
| H366F | H366F-F | GCCCAGCTCTTCGGCGGGTGCG |  |
|  | H366F-R | GCACCCGCCGAAGAGCTGGGCG |  |
| W245F | W245F-F | TTCCTGCTGTTCTTCGGCTGGT |  |
|  | W245F-R | CCAGCCGAAGAACAGCAGGAAG |  |
| H199F | H119F-F | CGGCGTCGTCTTCATGGTCGGC |  |
|  | H119F-R | CCGACCATGAAGACGACGCCG |  |
| W166F | W166F-F | GTCCCACTTCATCTGGTCCGCC |  |
|  | W166F-R | CGGACCAGATGAAGTGGGACAC |  |
| Y38F | Y38F-F | GTGGACGCGACGTTCCTGCTC |  |
|  | Y38F-R | GAGAAGAGCAGGAACGTCGCG |  |
| Y160F | Y160F-F | CCGGGTTCGTCTTCCCGGTGG |  |
|  | Y160F-R | GACACCACCGGGAAGACGAAC |  |
| F251S | F251S-F | GCTGGTACGGATCCAACCCCGG |  |
|  | F251S-R | GACCCGGGGTTGGATCCGTAC |  |
| M49A | M49A-F | CGTGTTCGCCGCGCAGCTCGGG |  |
|  | M49A-R | CGAGCTGCGCGGCGAACACGAG |  |
| Y84F | Y84F-F | GGCGCTCTTCTTCTACCTCTTC |  |
|  | Y84F-R | GCCGAAGAGGTAGAAGAAGAG |  |
| W173F | W173F-F | GCCGATGGGTTCGCCTCTGCCT |  |
|  | W173F-R | GGCAGAGGCGAACCCATCGGC |  |
| F127S | F127S-F | CCAGTGGGCCTCCGCCATCGC |  |
|  | F127S-R | GGCGGCGATGGCGGAGGCCCA |  |
| H410F | H410F-F | GCTCGCCGCGTTCGTCATCCAG |  |
|  | H410F-R | TGGATGACGAACGCGGCGAGCA |  |
| R142K | R142K-F | CCATCGCCGAGAAGACGCAGT |  |
|  | R142K-R | CGAACTGCGTCTTCTCGGCGA |  |
| D358N | D358N-F | CCTCAAGTTCGACAACCCGCTC |  |
|  | D358N-R | GCCTCGAGCGGGTTGTCGAAC |  |
| N252A | N252A-F | TACGGATTCGCCCCCGGGTCGT |  |
|  | N252A-R | GACCCGGGGGCGAATCCGTACC |  |
| OsAMT1;1 | OsAMT1;1-F | GGGGACAAGTTTGTACAAAAAAGCAGGCTTCGGTACCatggcgacgtgcgcggcgga |  |
|  | OsAMT1;1-R | GGGGACCACTTTGTACAAGAAAGCTGGGTGACTAGTcacttggttgttgctgttgg |  |

**Supplemental Table 2. qRT-PCR and RT-PCR primers used in this study.**

| **Primer name** | **Primer sequence**  **Forward (5'-3')** | **Primer sequence**  **Reverse (5'-3')** |
| --- | --- | --- |
| qUbiquitin | CAAGATGATCTGCCGCAAATGC | TTTAACCAGTCCATGAACCCG |
| qAMT1;1 | GAATAAATTATCCAATTAAC | CAAGTGGCTCTCCAACATTC |
| qAMT1;2 | GGATCCATGGCAACGTGCGCGGATA | AAGCTTCTAGACATGGCCTCCCATCT |
| qACO2 | TCAATGGCTACCACGTTAGATG | GATGTGACAGCCAAGAATTTCA |
| qACO3 | CGCCGCCGAGGTCGTCCACG | GCCCGTTACACACACTTGAG |
| qEIN2 | AACTGCGGAGACGACTGCAT | AGGATGCCCTGAAGACGGTT |
| qERS1 | TCATGGTTCTGATGCTTCCA | TGCTCCATTAGCAGATCACG |
| qETR2 | GTTCGTCATCCAGTCGGAGA | GAACTGAAGGGCAAGCATGA |
| qEIL1 | ACAATGCCACGATCATGGAG | TCAGTAGTACCAATTCGAGC |
| qEIL2 | CGCCGCACATCTTCGAGCCA | TCAGTAGAACCAGTTGGATCCG |
| qERF1 | CAGTGAAGCAAGCAAACCAA | GCTTATCGCGTTTGCAATTT |
| qERF2 | GTGGACCAGATGATCGAGGAG | CCAGAACTCACTGTGACCAA |
| RT-GDCi | GATGAGTCCGTCCACTCCAC | CTCGTCGACCATATCCACGG |
| RT-*AtAMT1;3* | GAAGGCCATATGGACTATTTATGGG | CGAGGAGGAGTAGCTGATCGAGG |

**Supplemental Table 3.** Primers used in this study for the construction of plants expressing *AtAMT1;3* *T464D-A141E* driven by *AMT1;1* endogenous promoter.

| **Primer name** | **Primer sequence (5'-3')** |
| --- | --- |
| Amtrac F-SalI | gtcgacATGTCAGGAGCAATAACATG |
| Amtrac R-BglII | agatctTCAAACGCGAGGAGGAGTAGC |

**Supplemental Table 4.** Primers used in this study for the construction of the overexpression vector.

| **Primers**  **name** | **Primer sequence**  **Forward (5'-3')** | **Primer sequence**  **Reverse (5'-3')** |
| --- | --- | --- |
| GDCi | CCAAGCTTATGGTGCTCTCCCACGG | GGGGTACCTTAGCAGACGCCGTTGGT |
| GS1;1 | CAAGCTTATGGCTTCTCTCACCGATCTC | GGGGTACCTCAGGGCTTCCAGATGATG |

**Supplemental Figures**


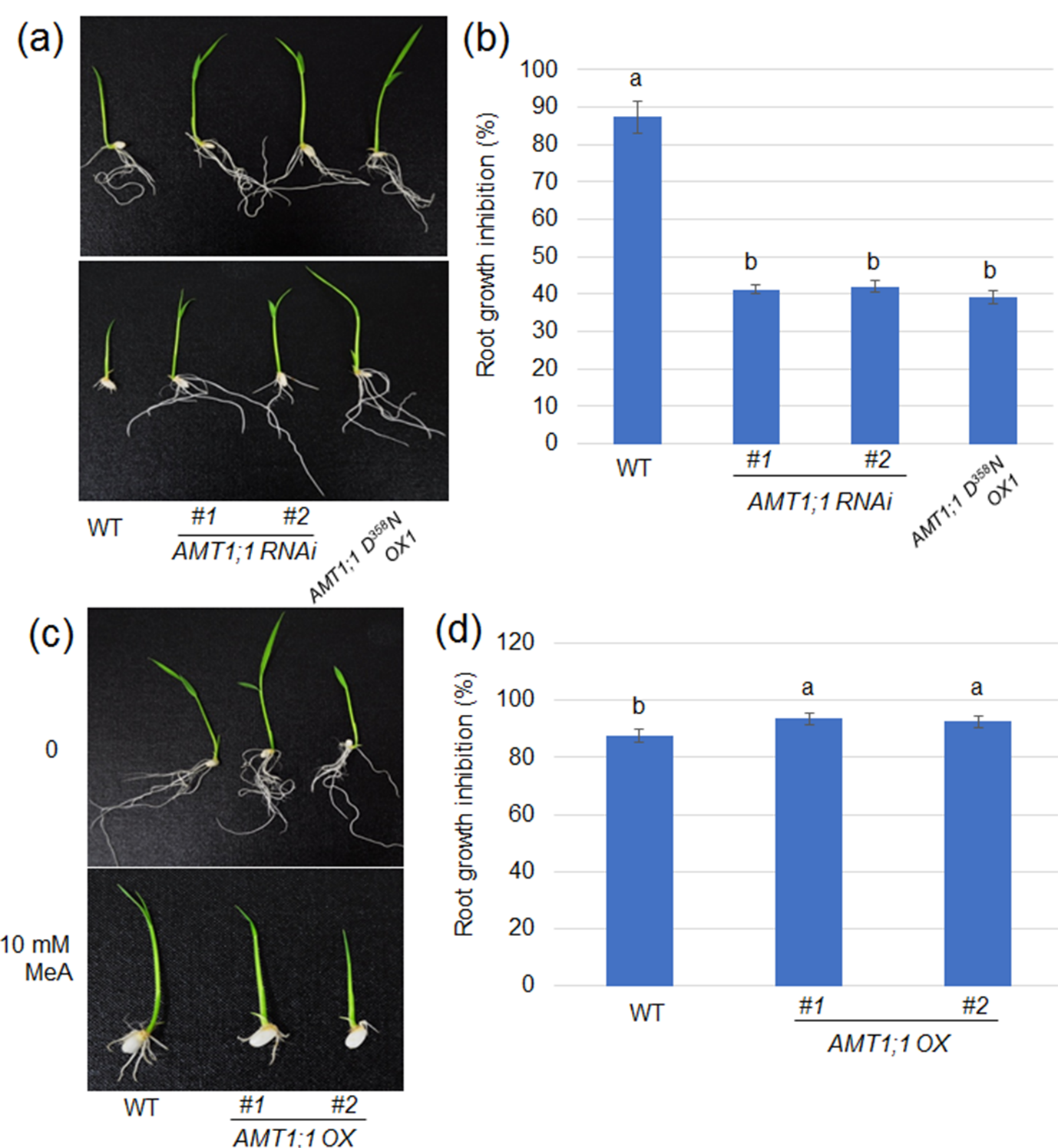


**Fig. S1** Sensitivity test of *AMT1;1 RNAi* and overexpression plants to methyl-ammonium (MeA). (a) Phenotypes of ten-day-old *AMT1;1 RNAi* (*Ri1*, *Ri2*) and wild-type plants grown on media with or without 10 mM of the toxic ammonium analog MeA. (b) Root growth inhibition ratio presented in (a). (c) Phenotypes of ten-day-old *AMT1;1 OXs* (*OX1*, *OX2*) and wild-type plants grown on media with or without 10 mM of the toxic ammonium analog MeA. (d) Root growth inhibition ratio shown in (c). Different letters represent significant differences (*P < 0.05*). The data are presented as means ± SD of 12 replicates.


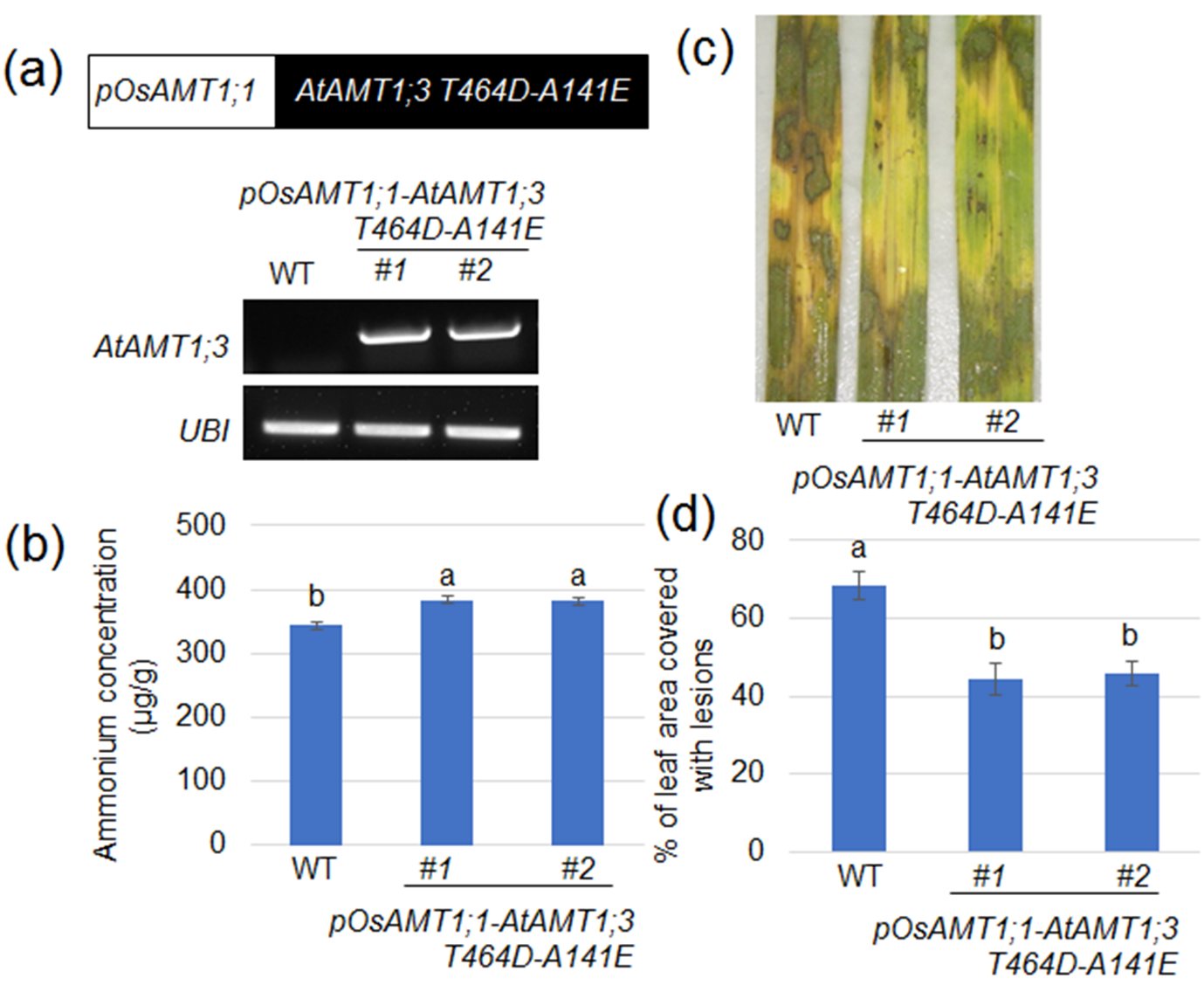


**Fig. S2** *AtAMT1;3* *T464D-A141E* expression promotes rice resistance to ShB. (a) RT-PCR detected heterologous expression of *AtAMT1;3* *T464D-A141E* in rice. *Ubiquitin* was used as the internal control. (b) NH_4_^+^ content in wild-type and *AtAMT1;3* *T464D-A141E* expressing plants (n>10). (c) The *AtAMT1;3* *T464D-A141E-*expressing and wild-type plants three days after inoculation with *R. solani* AG10IA. (d) Area ratio of leaves covered by lesions were measured in wild-type, *AtAMT1;3* *T464D-A141E-1,* and *AtAMT1;3* *T464D-A141E-2* plants (n>6). Different letters represent significant differences (*P < 0.05*). The data are presented as means ± SD.


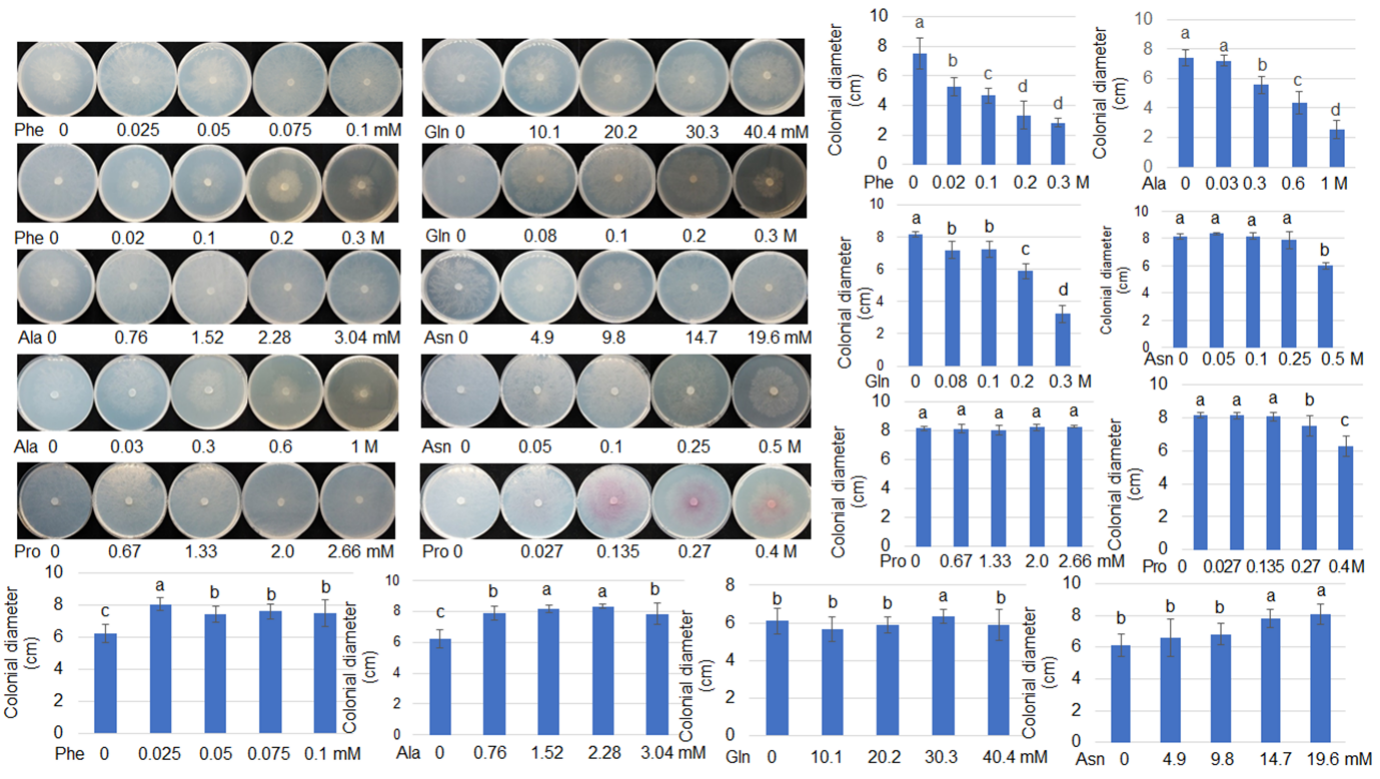


**Fig. S3** Verification of the effects of amino acids on *R*. *solani* growth. *R*. *solani* AG1-IA was cultured on a Czapek–Dox medium with the addition of different concentrations of amino acids (phenylalanine, alanine, glutamine, asparagine, or proline). PDA blocks (7 mm in diameter) covered with colonized hyphae were cut with a circular cutter and placed on the center of the media surface. These Petri dishes were cultured in a 37℃ incubator for 42 hours and the colony diameters (with original block diameter) were measured. The experiments were carried out for at least ten repetitions. The data are presented as means ± SE (n > 10). Different letters represent significant differences (*P < 0.05*).


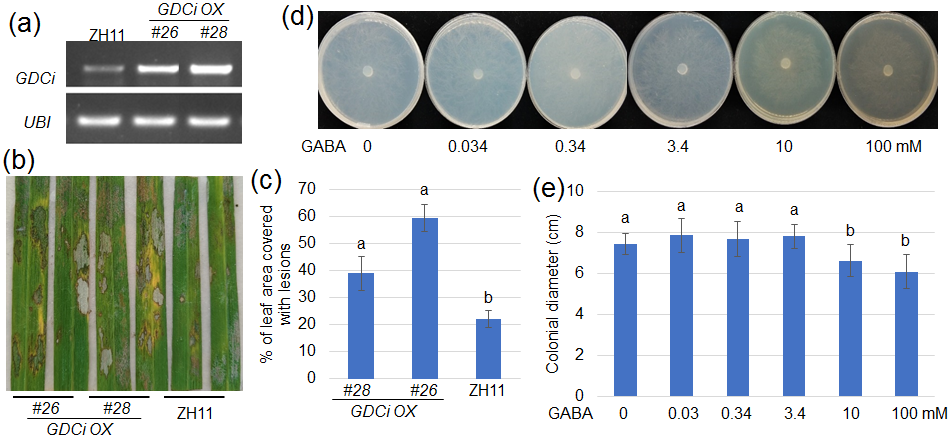


**Fig. S4** Identification of the effect of GABA on rice resistance against ShB. (a) Expression of glutamate decarboxylase (*GDCi*) measured in ZH11 and *GDCi OX* plants with RT-PCR. *Ubiquitin* was used as the reference gene. (b) Leaves from wild-type (ZH11) plants and *GDCi OX* plants were inoculated with *R*. *solani* AG1-IA and photographed after 48 hours. Ten leaves at the same growth stage from each line were analyzed and the inoculation experiments were carried out in triplicate. (c) The ratio of the lesion area to the whole tested leaf area was determined. The data are presented as means ± SE (n > 10). Significant differences at *P* < 0.05 are indicated by different letters. (d) *R. solani* AG1-IA was cultured on the Czapek–Dox medium with the addition of different concentrations of GABA. PDA blocks (7 mm in diameter) covered with colonized hyphae were cut with a circular cutter and placed on the center of the media surface. These Petri dishes were cultured in a 37℃ incubator for 42 hours and (e) the colony diameters (with original block diameter) were measured. The consistent experiments were carried out for at least ten repetitions. The data are presented as means ± SE (n > 10). Different letters represent significant differences (*P < 0.05*).


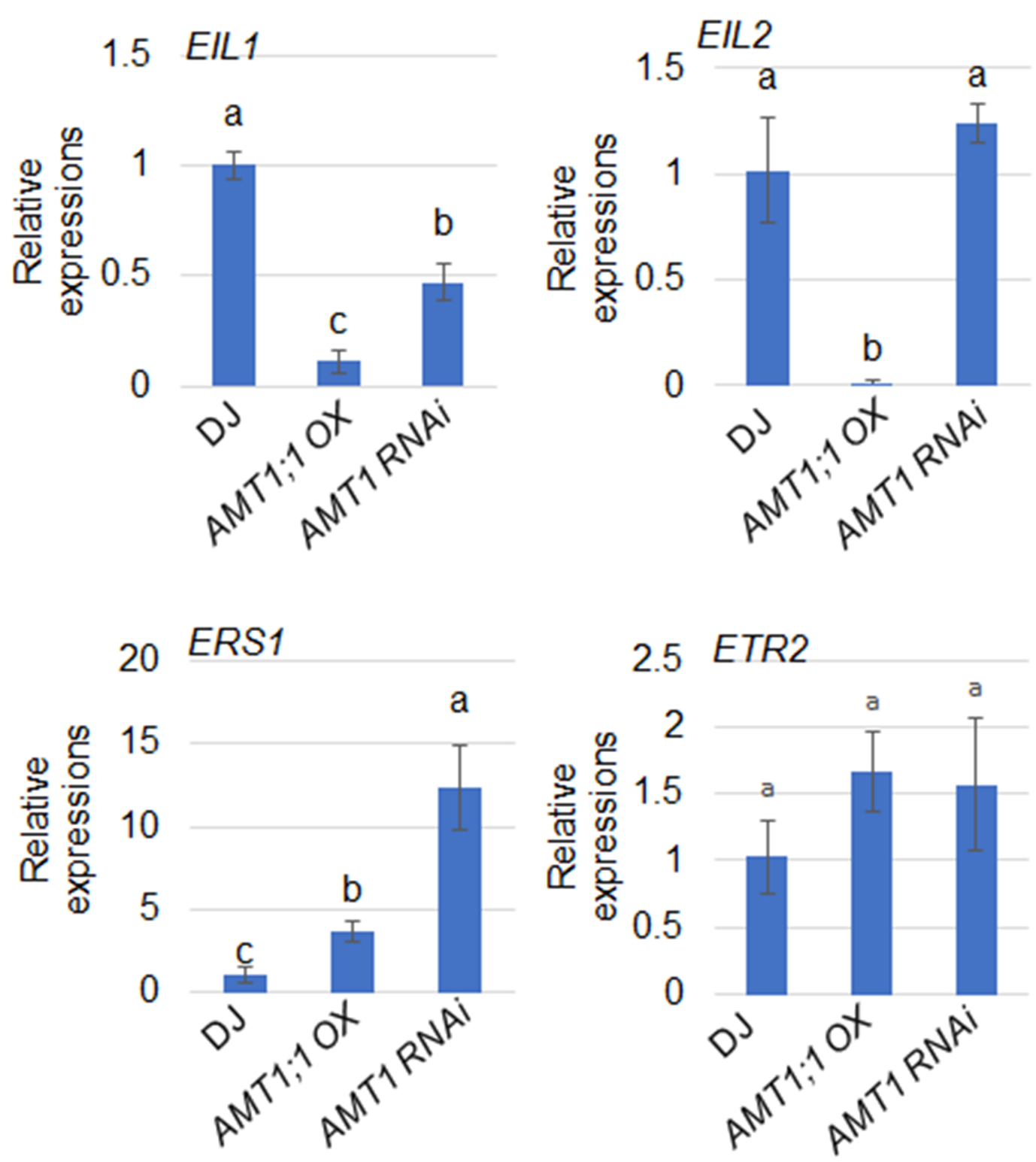


**Fig. S5** Expression levels of ethylene signaling genes under HN conditions in wild-type, *AMT1;1 OX,* and *AMT1;1 RNAi* plants were quantified by qRT–PCR. The relative expression levels of *EIL1*, *EIL2*, *ERS1*, *ETR2* in wild-type (DJ), *AMT1;1 OX,* or *AMT1 RNAi*plants cultured under HN conditions (300 kg·ha^-1^). *Ubiquitin* mRNA levels were used for the normalization of samples. Error bars represent means ± SE (*n* = 3). Different letters represent significant differences (*P < 0.05*).
